# MicroRNA-147 Induces a Mesenchymal-To-Epithelial Transition (MET) and Reverses EGFR Inhibitor Resistance

**DOI:** 10.1371/journal.pone.0084597

**Published:** 2014-01-15

**Authors:** Chang Gong Lee, Susan McCarthy, Mike Gruidl, Cindy Timme, Timothy J. Yeatman

**Affiliations:** 1 Gibbs Cancer Center & Research Institute, Spartanburg, South Carolina, United States of America; 2 H. Lee Moffitt Cancer Center & Research Institute, Tampa, Florida, United States of America; University of Central Florida, United States of America

## Abstract

**Background:**

The epithelial-mesenchymal transition (EMT) is a key developmental program that is often activated during cancer progression and may promote resistance to therapy. An analysis of patients (n = 71) profiled with both gene expression and a global microRNA assessment (∼415 miRs) identified miR-147 as highly anti-correlated with an EMT gene expression signature score and postulated to reverse EMT (MET).

**Methods and Findings:**

miR-147 was transfected into colon cancer cells (HCT116, SW480) as well as lung cancer cells (A-549). The cells were assessed for morphological changes, and evaluated for effects on invasion, motility, and the expression of key EMT markers. Resistance to chemotherapy was evaluated by treating cells with gefitinib, an EGFR inhibitor. The downstream genes regulated by miR-147 were assayed using the Affymetrix GeneChip U133 Plus2.0 platform. miR-147 was identified to: 1. cause MET primarily by increasing the expression of CDH1 and decreasing that of ZEB1; 2. inhibit the invasion and motility of cells; 3. cause G1 arrest by up-regulating p27 and down-regulating cyclin D1. miR-147 also dramatically reversed the native drug resistance of the colon cancer cell line HCT116 to gefitinib. miR-147 significantly repressed Akt phosphorylation, and knockdown of Akt with siRNA induced MET. The morphologic effects of miR-147 on cells appear to be attenuated by TGF-B1, promoting a plastic and reversible transition between MET and EMT.

**Conclusion:**

miR-147 induced cancer cells to undergo MET and induced cell cycle arrest, suggesting a potential tumor suppressor role. miR-147 strikingly increased the sensitivity to EGFR inhibitor, gefitinib in cell with native resistance. We conclude that miR-147 might have therapeutic potential given its ability to inhibit proliferation, induce MET, as well as reverse drug sensitivity.

## Introduction

The epithelial-mesenchymal transition (EMT) has been described as a cell-biological program that is required for the remodeling of cells and tissues during embryogenesis, during certain types of wound healing, and during the acquisition of malignant traits by carcinoma cells [1], [2].The epithelial-mesenchymal transition is a key developmental program that is often activated during cancer invasion, metastasis, and may promote resistance to chemotherapy.

MicroRNAs (miRNAs, or miRs) are noncoding mRNA sequences containing around 22-nucleotides that act as important regulators of gene expression. miRNAs can silence their cognate target genes by specifically binding and cleaving mRNAs or inhibiting their translation [3]. Some miRNAs have been shown to function as either tumor suppressors or oncogenes [4], [5]. miRNAs have recently been described as crucial regulators of the EMT and metastasis. The miR-200 family, which suppresses the EMT drivers ZEB1 and ZEB2, is selectively expressed in the sarcomatous component of metaplastic breast cancers [6]. Loss of the expression of any members of the miR-200 family may play a critical role in the repression of CDH1 by ZEB1 and ZEB2 during the EMT, thereby enhancing migration and invasion during cancer progression. Ectopic expression of the individual members of the miR-200 family, as clusters, or altogether hinders EMT progression in TGFβ-treated NMuMG cells [7], suggesting that they are both fundamental markers and powerful regulators of the EMT process [6], [8]. Additional miRs, such as miR-655 was also found to suppress EMT [9].

Unraveling the miRNA-mediated effects on EMT/MET, and their upstream and downstream targets is likely to reveal novel biomarkers for the advanced stages of cancer, improve prognosis and reveal new opportunities for therapeutic intervention [10].

While few published studies of miR-147 exist, an endogenous negative-feedback loop was recently reported, in which the stimulation of Toll-like receptors induced miR-147 in order to prevent excessive inflammatory responses [11]. Additional studies identified and verified three miRNAs (miR-124, miR-147 and miR-193a-3p) to be novel potential tumor suppressors that co-target EGFR-driven cell-cycle network proteins and inhibit cell-cycle progression and proliferation in breast cancer [12].

We recently reported an unsupervised, global gene expression analysis (Affymetrix GeneChip) of 250 frozen human colorectal cancer specimens (CRC) identified two intrinsic subpopulations of patients that were characterized by a signature strongly linked to the process of the epithelial-mesenchymal transition (EMT) [13]. A subset of 70 of these CRC patients were profiled with a global microRNA (∼415 miR) analysis to identify the specific miRs that were highly-correlated with the EMT signature score. The miR-200 family and miR-147 were among the miRs most highly anti-correlated with the EMT signature (Table S1). Therefore, miR-147 and other potential miRs may reverse the EMT process, moving cells from a mesenchymal to an epithelial phenotype (MET).

The current study was undertaken to understand the effects of miR-147 on the cell morphology, EMT/MET, the response to EGFR inhibitor therapy, and the potential downstream genes potentially regulating these processes.

## Materials and Methods

### Cell Culture

Colorectal cancer cell lines HCT116, SW480 and lung cancer cell line A549 were obtained from the ATCC (Manassas, VA) and cultured in RPMI 1640 medium (Cellgro) supplemented with 10% fetal calf serum.

### Invasion and proliferation assays

Matrigel-coated inserts (BioCoat Matrigel Invasion Chamber; Becton Dickinson) were used for the cell invasion assays. The floated died cells were discarded, only the attached cells were collected and seeded in the upper chambers (5×10^4^). FBA (5%) was added to the lower chambers. The cells in the upper chambers were allowed to invade for 16 hours. The inserts in the upper chambers were removed for analysis. Cells on the inserts were fixed in 90% ethanol, stained with 0.1% crystal violet blue, and washed with PBS buffer. Non-invaded cells on the upper side of the inserts were wiped off with a cotton swab. Invaded cells were counted, and the results were presented as the mean ± the S.D. The assay was replicated at least two times. And so do the following other experiments apart from microarray assay.

### Cell proliferation

Cells were seeded in a 96-well plate at a density of 5×10^3^ cells/well. Twenty microliters of MTT solution (5 mg/ml in phosphate buffered saline, PBS) was added to each well and the plates were incubated for an additional 3–4 hr at 37°C. The plates were centrifuged at 200 *g* for 5 min, the medium was aspirated from each well, and 180 µl of DMSO was added to each well to dissolve the formazan. The optical density was measured at 562 and 630 nm with a Delta Soft ELISA analysis program interfaced with a Bio-Tek Microplate Reader (EL-340, Bio-Metallics, Princeton, NJ). Each experiment was performed in 4–6 replicate wells for each drug concentration and carried out 3 or 4 times independently.

### Transfection of microRNA precursors and inhibitors

All precursor microRNAs were ordered from Applied Biosystem (Invitrogen). Lipofectamine® RNAiMAX Transfection Reagent (Invitrogen) was used to transfect precursor miRs into HCT116, SW480, A549 cells according to manufacturer's instructions. The final concentration of each miRNA is at 20 nM. Total RNA and protein were collected for assay 3 days post-transfection or as indicated.

### Cell cycle analysis

Cells were harvested, washed twice in PBS, and fixed in 70% ethanol on ice for at least 30 min. Cells were then stained with propidium iodide solution (50 µg/mL propidium iodide, 50 µg/mL RNase A, 0.1% Triton-X, 0.1 mM EDTA). Cell cycles were analyzed by FACS based on their DNA content using a Flow Cytometer (BD Biosciences, Franklin NJ), or or guava easycyte HT.

### EMT assay

A549 cells were seeded at a density of 16×10^4^ cells/well (6-well plates) in RPMI+10% FBS. Cells were allowed to adhere for 1 day and 10 ng/ml of TGF-β1 (R&D SYSTEMS) was added. A549 cells were differentiated for 48 h and then used in EMT experiments.

### Western blotting and antibodies

Cell lysates that were prepared from cells using RIPA buffer supplemented with inhibitors were separated on 8–12% gradient SDS-polyacrylamide gels and transferred to PVDF membranes (Bio-Rad Laboratories). Membranes were incubated in blocking solution (5% skim milk in TBST) and incubated with primary antibodies, followed by incubation with the appropriate HRP-conjugated secondary antibody (Sigma). The primary antibodies used in this study were: anti-Akt, pAkt(S473), anti-CDH1, anti-Zeb1, anti-slug, (Cell Signaling, Beverly MA); anti-cyclin D1, anti-p27 (BD Pharmingen); anti-β-actin (Sigma).

### Quantitative real-time (qRT)-PCR

Total RNA was prepared from cells using RNeasy Plus Mini Kit according to the manufacturer's instructions. Samples were normalized to RNU44 for miRNAs and GAPDH for mRNAs (Applied Biosystems). The probes used for the Taqman Assays were ordered from ABI Company. A qRT-PCR was performed with a Bio-Rad iCycler single-color real-time detection system. All experiments were performed in triplicate.

### Microarray assay

To investigate the genes affected by miR-147, five independent transfection using precursor miR-147 or negative control miR were done with HCT116 cells. Total RNA were isolated using RNeasy Plus Mini Kit. Microarray assays were performed using Affymetrix GeneChips U133 Plus2.0 (Affymetrix, Santa Clara, CA). Affymetrix data have been submitted to GEO: NCBI tracking system16901253.

### Flow cytometry

HCT116, A549 cells were seeded in 6-mm culture plates at the density of 1×10^5^ cells per dish. After incubating for 24 h, cells were treated with 20 µM gefitinib for 48 h. Adherent and floating cells were both collected and resuspended in cold PBS for analysis. For apoptosis assay, cells were stained using the Annexin V-FITC Apoptosis Kit (BD Pharmingen, USA) and 7-AAD. Samples were analyzed on the FC 500 Flow Cytometry Systems (Beckman Coulter).

### Statistical Analysis

All data are presented as the mean ± S.D. All data are expressed as the average with 95% confidence intervals. The differences were determined using a two-sided student's *t* test with two-sample unequal variance type.

## Results

### miR-147 overexpression in human cancer cells caused a mesenchymal-to-epithelial transition (MET)

The 15 microRNAs (Table S1) with the strongest correlations to the high EMT score (p<0.05) [13] were selected to validate their biological role. These miRs were transiently transfected into colon cancer cell lines HCT116 and SW480. The morphological changes associated with MET were assessed.

**Figure 1:**
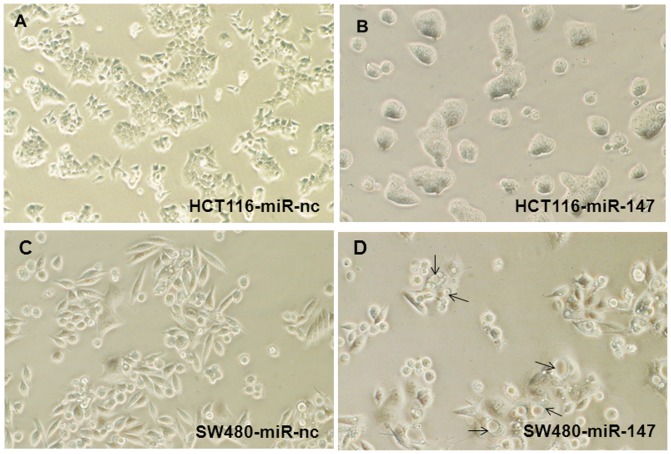
miR-147 induces a mesenchymal to epithelial transition in colon cancer cell lines. HCT116 and SW480 are colorectal cell lines. miR-147 induces the cells a phenotype change, from a speculated and loosely connected mesenchymal phenotype on the left (negative control) to a more tightly associated rounded epithelial phenotype. The precursor miR-147 and negative control miR were transiently transfected into the cells, the phenotype changes noted two days after transfection. (A) Negative control miR (miR-nc) transfected HCT116 cells show a speculated and loosely connected mesenchymal phenotype (Original magnification of 100×). (B) miR-147 induces the HCT116 cells a phenotype change to a more tightly associated rounded epithelial phenotype. (C) miR-nc transfected SW480 cells are in loosely connected, spindle mesenchymal phenotype. (D) miR-147 transfected induced SW480 cells a more tightly associated rounded epithelial phenotype and many cells with big vacuoles (arrows). (B–D: Original magnification of 200×).

The endogenous levels of miR-147 in HCT116 and SW480 cells were examined using RT-PCR. The amount of miR-147 in the two cell lines was too low to be detected (data not shown). Morphological changes were clearly observed two days after transfection with miR-147. The expression of miR-147 induced an obvious phenotype change in the HCT116 and SW480 cells (Figure 1) from the speculated and loosely connected mesenchymal phenotype in the negative control miR (miR-nc) transfected cells on the left (Figure 1A, C and Figure S1A, S2A) to the more tightly associated, rounded, epithelial phenotype of miR-147 transfected cells on the right (Figure 1B, D and Figure S1B, S2B). These observations indicate that the ectopic expression of miR-147 induce MET in colon cancer cells.

### miR-147 repressed cell invasion and proliferation, induced cell arrest at G1

**Figure 2:**
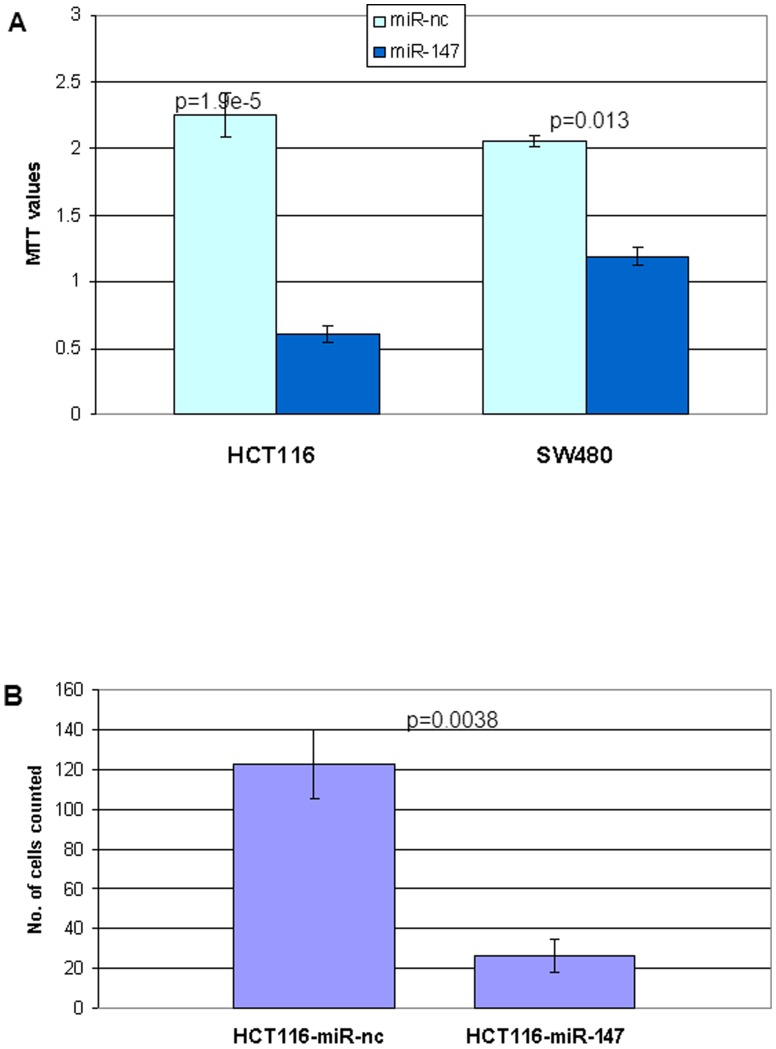
miR-147 reverses mesenchymal phenotype. (A) Ectopic expression of miR-147 inhibits cell proliferation. MTT assay showed that the OD at 490 nm values of miR-147-transfected HCT116 and SW480 Cells decreased significantly compared to negative control miR transfected cells, (p = 1.9e-5, and p = 0.013 respectively). (B) miR-147 inhibits invasion. Cell invasion was done by Matrigel chamber. HCT116 and SW480 Cells were transfected with miR-147 and negative control miRNA. After 48 h of transfection, floated died cells were washed away, live cells were put into Matrigel inserts for invasion assay as illustrated in Materials and Methods. The data show means ± SD of the numbers of invading cells from three independent experiments. (p = 0.0038).

Proliferation and invasion are two characteristic aggressive properties of cancer cells. The EMT process is associated with enhanced cellular motility and invasiveness. The observation that miR-147 induced cells to undergo MET, suggested that it may affect cell proliferation and invasiveness. A 3-[4, 5-dimethylthiazol-2-yl]-2, 5-diphenyltetrazolium bromide (MTT) assay was used to investigate the effect of miR-147 on cell proliferation. Figure 2A demonstrates that there was a significant decrease in the MTT value in miR-147 transfected HCT116 and SW480 cells on day 3, in comparison to the negative control miR transfected cells (p = 1.9E5 and 0.013 separately). This finding indicated that miR-147 inhibited cell proliferation.

A Matrigel invasion chamber assay was used to measure the cell invasive capability. Figure 2B shows that the number of cells that invaded the Matrigel. HCT116-miR-147cell number was about 5-fold less than that of the microRNA transfected negative control cells (p = 0.0038). To avoid the possibility that apoptosis influenced invasion, we used only attached (live) cells in the invasion assay. Floating cells were removed prior to trypsinizing attached cells.

**Figure 3:**
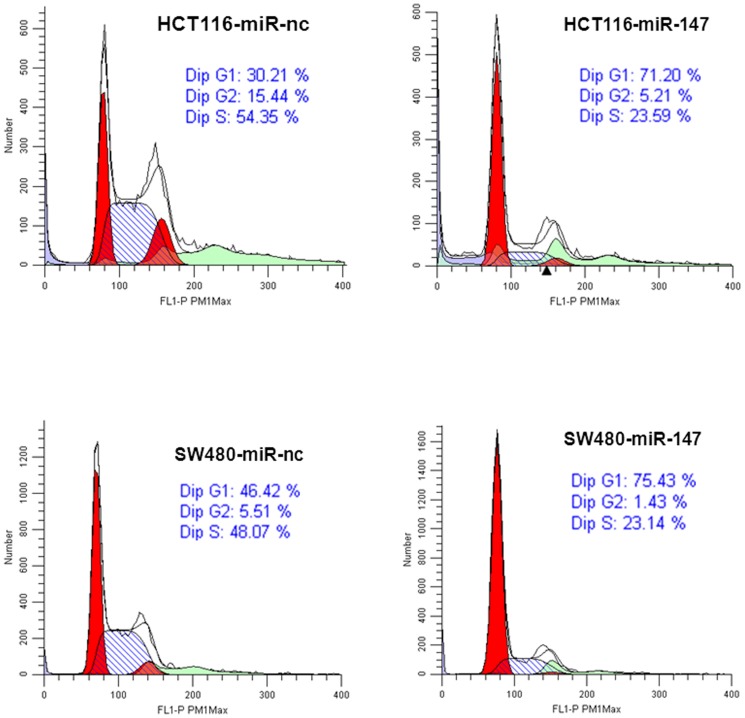
miR-147 affects cell cycle, induces cell arrest in G1 phase. The negative control miR transfected HCT116 and SW480 cells show a standard cell cycle pattern (left), while the miR-147 transfected cells, show a dramatic reduction in S Phase (right). Cells transiently transfected with miR-147 and negative control miR, after 3 d post-transfection, DNA content was measured by flow cytometry to determine cell cycle fractions. Representative flow cytometric histograms of cells shown from three independent experiments. PI, propidium iodide.

The effect of the ectopic expression of miR-147 on the cell cycle was examined to determine the mechanisms by which miR-147 mediates growth suppression. miR-147 or a negative control miR was transfected into HCT116 and SW480 cells. miR-147 transfected cells show a dramatic increase in the G1 phase and decrease in S-phase compared to the negative miR transfected cells with both HCT116 and SW480 cells (Figure 3).

### miR-147 regulated key EMT markers and p27, cyclin D1

**Figure 4:**
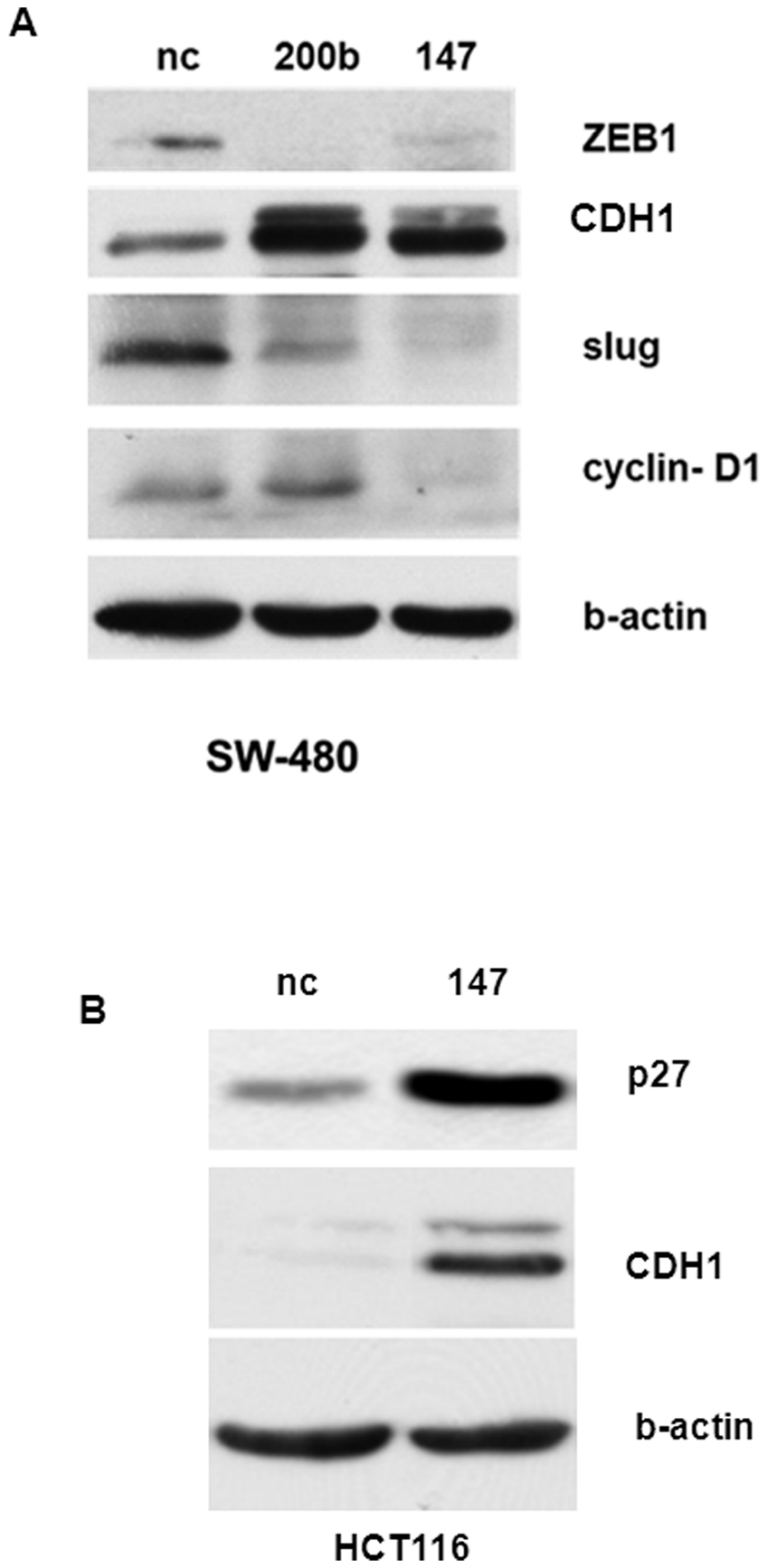
miR-147 affected the expression of EMT markers and cell cycle regulators. (A) miR-147 upregulates CDH1 and downregulates ZEB1, Slug and cyclin D1 proteins in colon cancer cell line SW480. (B) Ectopic expression of miR-147 upregulates CDH1 and p27 in HCT116 cells. The cells were collected after 3 days of miRs transfection, and lysate with RIPA buffer, then subjected to Western blotting assays.

The previous experiments demonstrated that the ectopic expression of miR-147 induced a visible morphological change in human cancer cells from mesenchymal to an epithelial phenotype (Figure 1), and caused cell arrest in G1 phase. These observations lead to the question of how miR-147 induced MET and arrested the cell cycle. Further experiments examined the expression of some key EMT markers and cell cycle related proteins. Figure 4 shows that the ectopic expression of miR-147 in HCT116 cells significantly upregulated the expression of CDH1 and p27. In addition, miR-147 upregulated CDH1, o27 expression and downregulated ZEB1, Slug and Cyclin D1 expression in SW480 cells. miR-200b, an EMT inhibitor, served as a positive control. Therefore, miR-147 induced a MET through upregulating CDH1 and downregulating ZEB1, Slug. In addition, miR-147 caused G1 arrest through upregulation of p27 and downregulation of cyclin D1.

### miR-147 reverses TGF-β1 induced EMT

TGFβ is a master regulator of EMT, and appears to play a dominant role in directly activating ZEB1 transcription factors. The addition of TGF-β to culture is a convenient way to induce EMT in epithelial cells.

**Figure 5:**
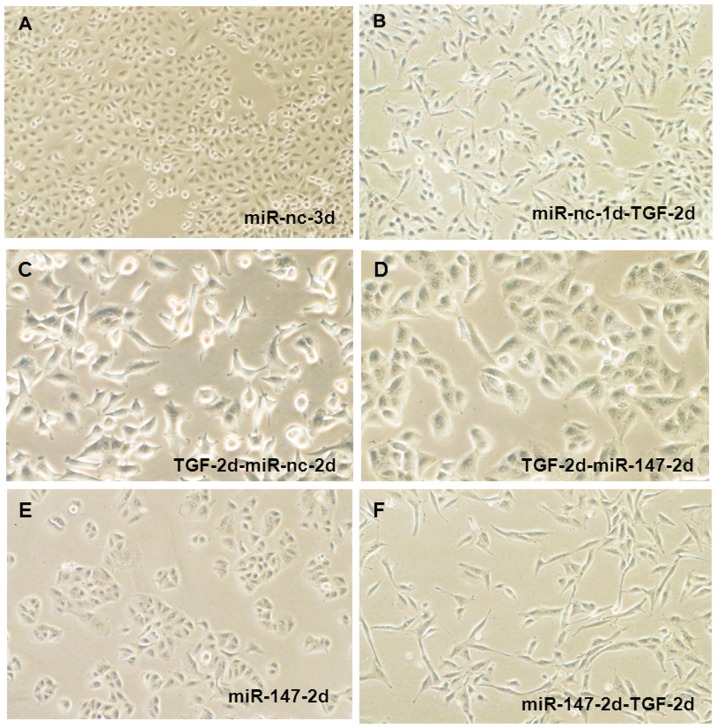
miR-147 reverses TGF-β1 induced EMT and affected ZEB1/CDH1. (A) A549 cells were transfected with negative control miR for 3 days. (B) A549 cells transfected with miR-nc for 1 day then were treated with 10 ug/ml TGF-β1 for 2 days and the cells phenotype changes form epithelial state to mesenchymal type. (C) The A549-miR-nc cells treated with TGF-beta1 for 2 days were then transfected with miR-nc for 2 days. (D) The A549-miR-nc cells treated with TGF-β1 for 2 days were then transfected with miR-147 for 2 days. (E) A549 cells were transfected with miR-147 for 2 days as a kind of control. (F) The A549 cells transfected with miR-147 for 2 days were then treated with TGF-β1 for 2 days. (Figures 6A, B, E, F: original magnification of 100×, C & D: 200×).

Normal A549 cells show uniform cobblestone morphology with intimate cell-cell contact (Figure 5A). TGF-β1 treated A549 cells lost the intimate cell-cell contact, and adopted a more elongated morphological shape (Figure 5B). The negative miR or miR-147 was transfected into TGF-β treated A549 cells to determine whether it could reverse the TGF-β induced EMT. The negative miR did not change the cell phenotype (Figure 5C), miR-147 caused mesenchymal A549 cells induced by TGF-β to undergo a phenotypic change from the mesenchymal state to a clear epithelial state (Figure 5D), which is similar to miR-147 transfected cells alone (Figure 5E). In addition, further treatment to the miR-147 transfected cells with TGF-β induced the cells to revert back to the mesenchymal phenotype, characterized by spindle-shaped cells (Figure 5F).

**Figure 6:**
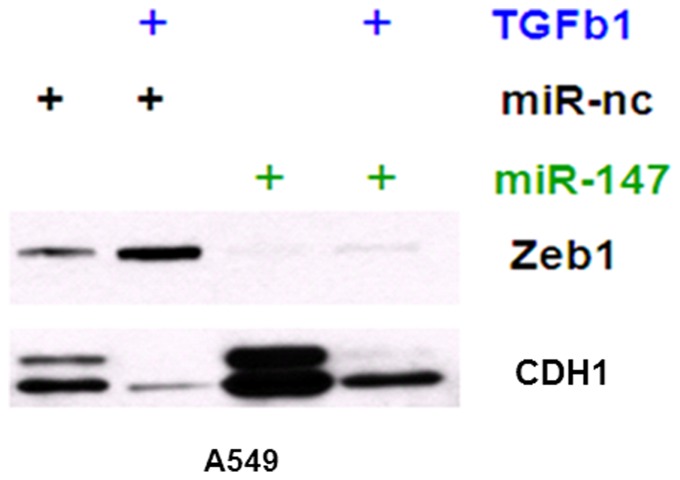
A549 cells were treated with TGF-beta1 first, then transfected with miR-147 or negative control miR, then treated with TGF-beta1, collected cells and did Western blot to check the expression of EMT marker proteins CDH1 and ZEB1.

Further examination of the EMT marker proteins and their response to miR-147 revealed that TGF-β treatment increased ZEB1 activity and inhibited CDH1 expression in miR-nc transfected A549 cells as expected. However, the ectopic expression of miR-147 increased the expression of CDH1 and decreased that of ZEB1 (Figure 6), which is consistent with its role in inducing MET. These data suggest that miR-147 and TGF-β may function reciprocally, and that there is an autocrine TGF-β/miR-147 signaling network that regulates the epithelial-mesenchymal transition. TGF-β-induced EMT can be suppressed by miR-147, independently of other miRs such as the miR-200 family members, through the translational inhibition of ZEB1 and the upregulation of CDH1 in cancer cells.

The cell cycle was also examined with miR-147 or negative control miR transfected A549 cells. The negative control miR transfected cells showed the standard cell cycle progression, from the G1 to the S-phase, and to the G2 phase (Figure S3A), while the miR-147 transfected cells show a dramatic increase in the G1 phase (from 54% to 81%), and a reduction in the S Phase (from 37% down to 10%) (Figure S3B) compared to the negative miR transfected cells.

### miR-147 reverses drug resistance associated with the “epithelial” EGFR inhibitor, gefitinib

Gefitinib is an oral, reversible, inhibitor of epidermal growth factor receptor (EGFR)-associated tyrosine kinase, which exhibits its antitumor activity by the blockade of EGF receptor-associated tyrosine kinase activity. The vast majority of these tumors ultimately become resistant to the drug treatment with both intrinsic and acquired resistance to EGFR inhibitors.

**Figure 7:**
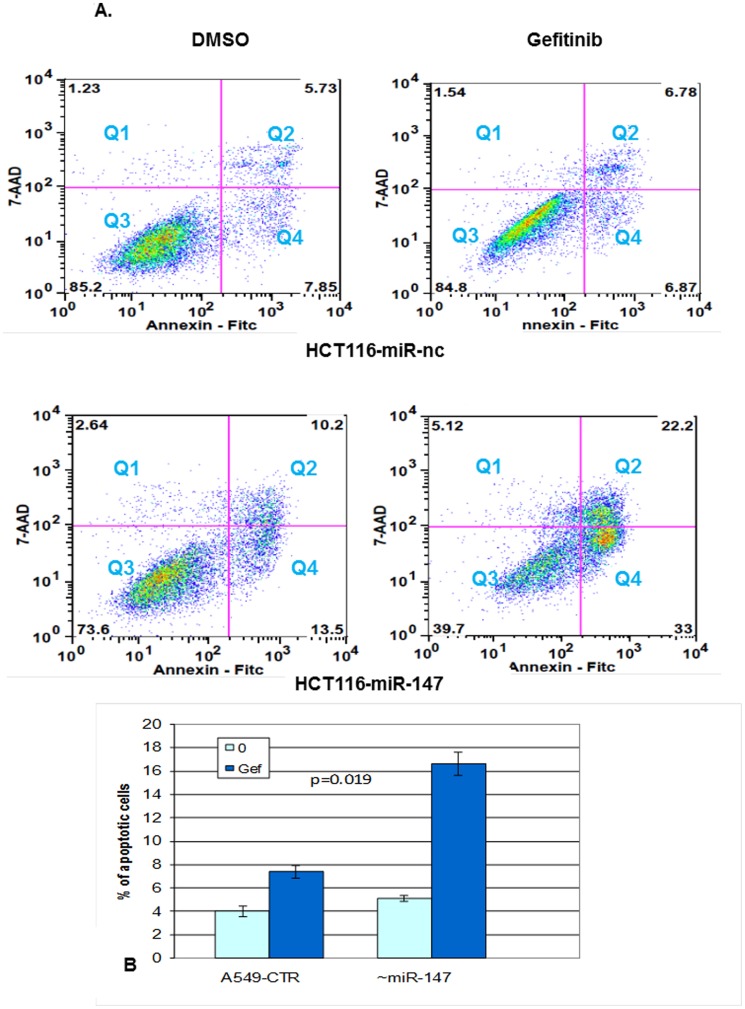
miR-147 recovered cell sensitivity to EGFRi gefitinb. (A) miR-147 induced cell sensitivity to EGFRi in the natively resistant gefitinb HCT116 cell line. The concentration of gefitinib was 20 uM and treatment for 24 hours. Cell apoptosis is measured by flow cytometry analysis of Annexin V-FITC double-labeled cells transfected with miR-147 and miRNA control. Flow cytometry profile represents Annexin V-FITC staining in *x* axis and 7-AAD in *y* axis. Dual staining of cells with Annexin V-FITC and 7-AAD enabled categorization of cells into four regions. Region Q1 shows the necrotic cells, Q2 shows the late apoptotic cells, Q3 shows the live cells, and Q4 shows the early apoptotic cells. The experiment was repeated more than three times and data represent the average of the early apoptotic and late apoptotic cells. (B) miR-147 similarly recovered A549 cells' sensitivity to EGFRi gefitinb. p = 0.019 between gefitinib treated miR-147 and negative control miR transfected A549 cells. The transfection and flow cytometry analysis were done as in Figure 6A.

The HCT116 and SW480 colon cancer cell lines were treated with gefitinib (10, 20, 30 uM). A significant number of SW480 cells floated and died after 24 h treatment; however, most of the HCT116 cells survived, confirming that HCT116 cells are intrinsically resistant to gefitinib. Then we transfected miR-147 into HCT116 cells and see if it had effect on cell's resistance to gefitinib. HCT116-miR-147 cells were treated with Gefitnib for 24 hours after transfection to determine miR-147's effect on cells' resistance to Gefitinib. Figure 7A shows that miR-147 induced a substantial increase in apoptosis in cells treated with gefitinib in comparison to control cells that transfected with a non-coding control miR.

miR-147 was also transfected into A549 cells, which were subsequently treated with gefitinib, and the result showed a similar trend but the apoptotic rate was lower than that in HCT116-miR-147 cells (Figure 7B). This supports the hypothesis that miR-147 reverses cell resistance to EGFR inhibitor gefitinib following a mesenchymal to epithelial transition.

### Genes affected by miR-147

miR-147 has shown the ability to reverse the EMT and convert EGFRi-resistant cells to sensitive cells. In order to further understand the mechanisms responsible for these activities. The genes affected by the ectopic expression of miR-147 in colon cancer cells were analyzed using a global gene expression survey (Affymetrix, Santa Clara, CA). The expression of more than 1700 genes were downregulated >2.0 fold in association with the expression of miR-147 compared to the control and, that of 1600 genes were upregulated over 2.0 fold, in comparison to negative control miR transfected HCT116 cells (Table S2, Affymetrix data file).

Table 1 lists several EMT and cell cycle related genes. TGF-β1 levels were reduced 2.4 fold in HCT116-miR-147 cells compared to negative control. miR-147 also downregulated AKT2, CDK1, CDK4, cyclin F which regulate the cell cycle. EMP1 was inhibited more than 6 fold, and this expression was related to acquisition of gefitinib resistance.

**Table 1:** Part of genes affected by miR-147 in HCT116 cells.

Genes	Fold changes	Exp in miR-nc cells	Exp in miR-147 cells
Stat3	+5.25	1790.8	1790.8
TP53I3	+12.09	8265.1	8265.1
EMP-1	−6.44	448.6	448.6
Cyclin D1	−2.60	2672.4	2672.4
Cyclin F	−2.55	598.2	598.2
CDK4	−2.14	3827.6	3827.6
CDK1	−3.79	1432.5	1432.5
TGF- β1	−2.40	243.9	243.9
MET	−3.09	91.7	91.7
Akt2	−2.94	411.6	411.6
TWIST1	−1.95	138.6	138.2

The analysis done by Affymetrix GeneChip U133 Plus2.0 platform. RNA from 5 isolated transfections of HCT116 cells transfected with miR-147 or miR-nc.

### miR-147 repressed Akt phosphorylation

**Figure 8:**
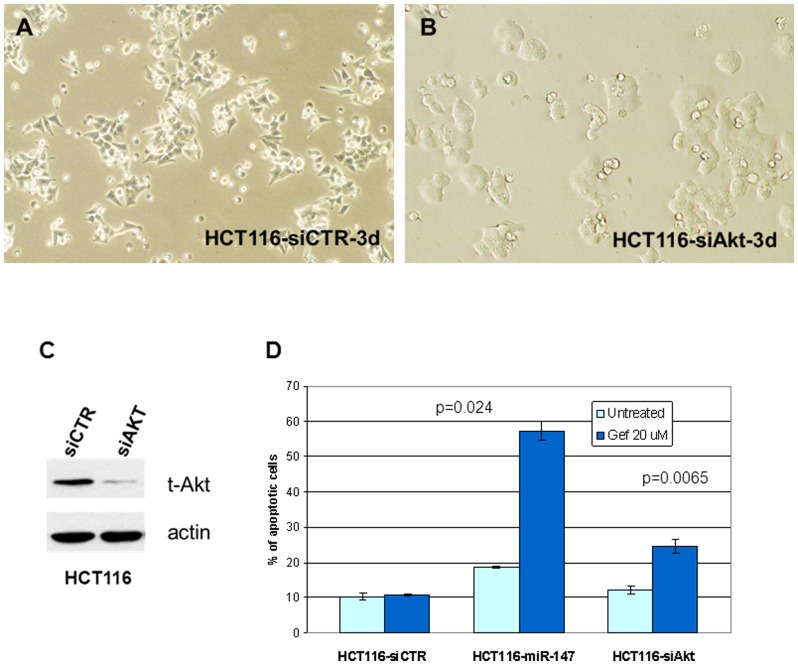
Knockdown of Akt induces HCT116 cells a phenotype change. (A) HTC116 cells transfected with negative control siRNA (Original amplification of 100×). (B) HTC116 cells transfected with siRNA of Akt1/2. (Original amplification of 200×). (C) Knockdown of Akt: 20 nM siRNA was transfected into HCT116 cells and incubated for 3 days. Then cells collected for Western blotting. (D) Akt knockdown changed cell sensitivity to EGFRi gefitinib in HCT116 cell. p = 0.024 indicates the comparison between gefitinib treated siAkt and miR-147 transfected cells.

AKT plays a key role in multiple cellular processes. Activation of AKT promotes many of the processes critical to the malignant phenotype. Among the miR-147 down-regulated genes, AKT2 was down-regulated ∼3.0 fold, so we decided to investigate how miR-147 affects AKT, and the role of AKT in miR-147 induced MET and EGFR inhibitor sensitivity change. The expression of AKT was knocked down with siRNA directed at AKT1/2 in HCT116 cells (Figure 8C) to effectively mimic the effect of miR-147. AKT knockdown induced changes in the cellular phenotype that were similar to that observed with the miR-147 induced MET (Figure 8A and B. Ref. to Figure 1A and B, Figure S1). Gefitinib treated, AKT-knocked down HCT116 cells resulted in the recovery of some EGFRi sensitivity (i.e. induced apoptosis), but not to the degree that was accomplished with miR147 (Figure 8D).

**Figure 9:**
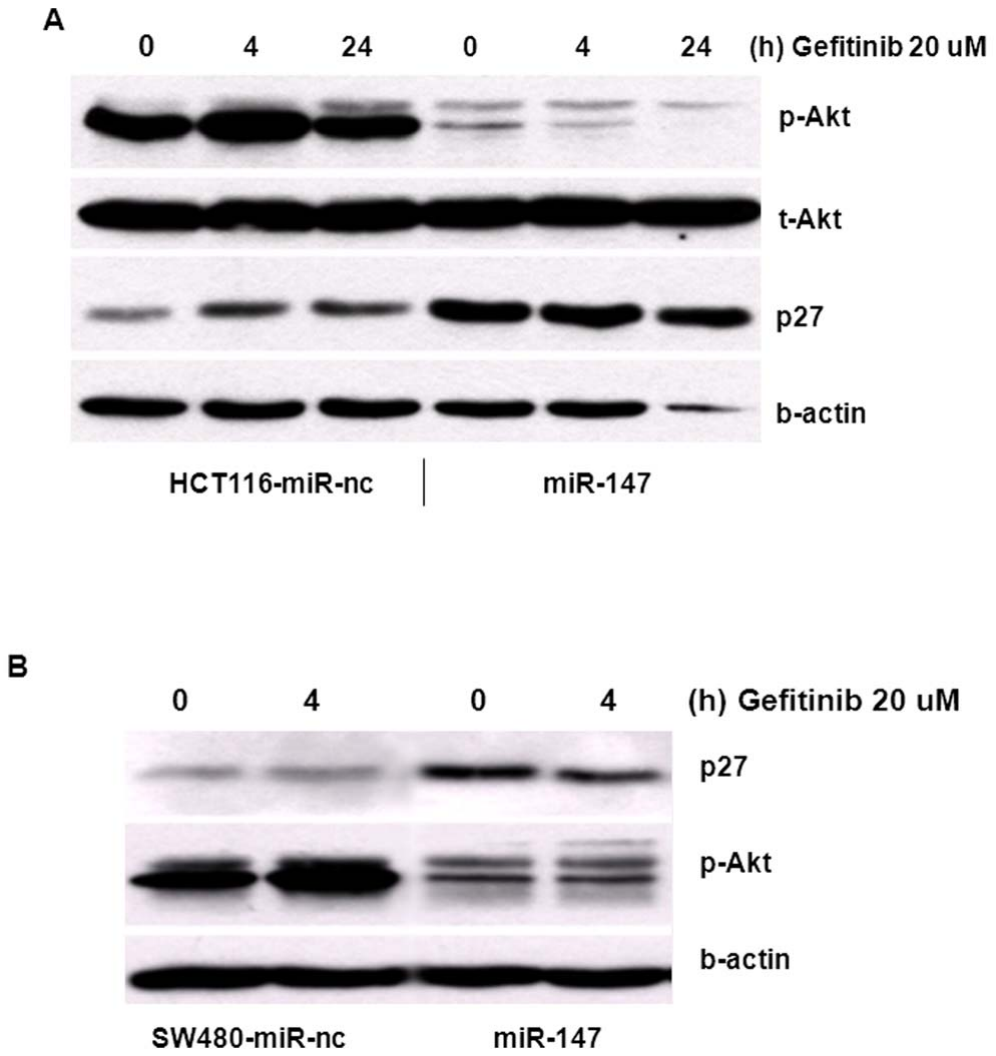
miR-147 inhibits Akt phosphorylation phosphorylation. The miRs were transfected into HCT116 and SW480 cells, gefitinib was added to miR-147 or negative control miR transfected after 2 days post transfection. The treatment for 4, 24 hours, then cells collected, lysates preparation were indicated in Materials and Methods, then checked the expression of proteins Akt (total and phosphorylated), p27, and β-actin as loading control.

Western blotting was performed to assess the expression and phosphorylation of Akt protein. Figure 9 demonstrates that miR-147 significantly repressed Akt phosphorylation in HCT116 and SW480 cells, but seemed to have no effect on the total Akt level. Gefitinib treatment did not affect the levels of Akt either in total or activated form in miR-nc transfected HCT116 cells. But in the sample of miR-147 transfected cells alone and in gefitinib treated cells, the phosphorylated Akt was substantially reduced. miR-147 upregulated p27 in both colon cancer cell lines HCT116 and SW480.

## Discussion

The EMT is a well-established phenotypic transformation that occurs during embryonic development. Recent studies have demonstrated that tumor cells utilize a process very similar to the development-associated EMT during tumor progression. Transition from an epithelial phenotype to a mesenchymal-like phenotype allows the cells to invade the surrounding stromal tissue, migrate into lymphatic or vascular tissue and metastasize to distant sites. An EMT results in increased metastatic behavior, drug resistance, cancer stem cell transformation, and poorer prognosis for a number of human cancers. Conversely, the mesenchymal to epithelial phenotype transition (MET) may allow circulatory metastatic tumor cells to recapitulate the primary tumor morphology and colonize distant organ sites such as the liver and lungs

This study demonstrated that the ectopic expression of miR-147 alone induced colon cancer cells to undergo MET, a process that can be reversed by treatment with TGF-B1. This process inhibited cell proliferation and invasion, induced cell cycle arrest at G1 and, restored the cells sensitivity to gefitinib, an inhibitor of EGFR tyrosine kinase. In addition, the current findings suggested the possibility of an endogenous miR-147/TGF-β signaling network that regulates the epithelial-mesenchymal transition that may be required for distant metastasis.

CDH1 and ZEB1 are key markers of the EMT. Loss of CDH1 expression is associated with poor prognosis in cancer patients and is considered to be a tumor suppressor [14], [15]. CDH1 is actively involved in maintaining epithelial characteristics. ZEB1 is an activator of the EMT and has crucial roles in tumor progression towards metastasis. ZEB1 and the miR-200 family members repress their respective expression in a reciprocal feedback loop. ZEB1 is a crucial promoter of metastasis and inhibits the expression of the miR-200 family, whose members are strong inducers of epithelial differentiation [16] [17]. The current study found that miR-147 significantly upregulated CDH1 expression and repressed ZEB1 expression, resulting in the reversal of EMT. Increasing evidence shows that the EMT is regulated by a balanced expression of ZEB factors and miR-200 family members, which are reciprocally linked in the ZEB/miR-200 feedback loop. The clarification of these points might lead to new therapeutic options for diseases such as chronic fibrosis and cancer [18]. In this report, miR-147 is a relatively poorly understood microRNA whose expression alone may induce cells to undergo MET. Our data suggest multiple biological effects linked to miR-147 likley through the concerted action of multiple target genes. For example, inhibition of cancer cell proliferation and invasion were seen, as well as induction of cell cycle arrest at G1 phase through up-regulation of p27, down-regulation of cyclin D1, and repression of Akt activation. Activation of Akt was previously shown to overcome cell cycle arrest in G1 [19].

TGF-β1 is a major inducer of the EMT in development, carcinogenesis, and fibrosis with different isoforms mediating various effects depending on specific cellular context [20]. TGF-β1 was first described as an inducer of EMT in normal mammary epithelial cells [21]. The addition of TGF-β1 to epithelial cells in culture is a convenient way to induce an EMT in various types of epithelial cells. Studies employing the cancer derived human alveolar epithelial cell line, A549, have confirmed the ability of alveolar epithelial cells to undergo an EMT *in vitro* [22]. TGF-β1 treatment induces A549 cells undergoing EMT and elicits a strongly mesenchymal phenotype [23]. In our study, miR-147 may induce such mesenchymal A549 cells back to epithelial state. Further treatment of these cells with TGF-β1 again induced the epithelial A549 cells back to mesenchymal phenotype. These results demonstrate that miR-147 alone may induce an MET, and also miR-147 and TGF-β1 may repress their respective functions. In addition, TGF-β1 increased the expression of ZEB1 while repressing that of CDH1; miR-147 had the opposite effects. These results are similar to those seen with the forced expression of the miR-200 family which is sufficient to block a TGF-β1-induced EMT [6]–[8].

TGF-β1 induces rapid activation of PI3K, Akt, mTOR complex 1 (mTORC1) and S6 kinase in cells that undergo EMT in response to TGF-β, leading to increases in protein synthesis, cell size, motility and invasion [24]. mTORC2 phosphorylates Akt on Ser473 [25], which, together with Akt phosphorylation on Thr308 by PDK1 in response to PI3K activation, confers full activity to Akt. In our study, ectopic expression of miR-147 inhibited TGF-β1 expression and reversed TGF-β1-induced mesenchymal cells back to the epithelial state; Conversely, miR-147 decreased Akt phosphorylation. In HCT116 cells, miR-147 decreased the expression of Akt2 both in mRNA and protein, but did not affect Akt1. In addition, knockdown of Akt1/2 induced HCT116 cells to change to an epithelial phenotype (Fig. 7B) similar to that induced by miR-147 (Fig. 1). miR-147 is known to target Akt2 and cyclin D1 as confirmed by Uhlmann et al [12]. Collectively,these data support that miR-147 induces MET by repressing TGF-β1 and decreasing Akt phosphorylation and subsequent activation.

The G1/S transition in the cell cycle is regulated by the CDK2/cyclin E complex. This transition can be inhibited by the cyclin dependent kinase inhibitor, p27 (Kip1). It is reported that the overexpression of p27^KIP1^ causes cell cycle arrest in the G_1_ phase [26]. There is recent evidence that high levels of p27^KIP1^ protein, induced by adenovirus vector, lead to growth arrest [27]. There is a significant correlation between p27^KIP1^ expression, cell cycle arrest and apoptosis [28]. The current study showed that miR-147 expression induced a G1 arrest in cells. Moreover, the ectopic expression of miR-147 leads to a significant elevation of p27, as revealed by a Western blot analysis. miR-147 down-regulates cyclin D1, which is essential for G1 progression [29], and is consistent with other report on miR-147 [12]. However, this is in contrast to miR-200 which promotes the expression of cyclin D1 protein. mir-200b directly reduced the expression of RND3 at the mRNA and protein levels, which thereby promoted expression of the downstream protein cyclin D1 and increased S-phase entry (a report that demonstrated that miR-200b increases cyclin D1 expression by targeting RND3 in HeLa cells) [30]. In addition, miR-147 also down-regulated other several genes like cyclin F, CDK1 and CDK4, which also affect the cell cycle (Table 1).

The EGFR has been strongly implicated in the biology of human epithelial malignancies, with therapeutic applications in cancers of the colon, head and neck, lung, and pancreas. The EGFR family members are common targets for cancer therapy. Small kinase inhibitor molecules have been tested for their potential as cancer therapeutics [31]. Gefitinib is an inhibitor of the EGFR tyrosine kinase (TKI) [32]. Gefitinib inhibits EGFR tyrosine kinase by binding to the adenosine triphosphate (ATP)-binding site of the enzyme [33]. Gefitinib is effective against colorectal tumor cells that express high levels of EGFR, and there is ongoing clinical evaluation of the efficacy of gefitinib in combination with CPT-11, in the treatment of colorectal cancers [34]. Despite the initial and often dramatic responses of EGFR-dependent lung tumors to the EGFR-specific tyrosine kinase inhibitors (TKIs), gefitinib and erlotinib, nearly all of these tumors develop resistance and relapse [35]. Almost all patients responsive to EGFR-TKIs show acquired resistance, which is a major clinical problem [23]. The EMT process is associated with both intrinsic and acquired resistance to EGFR-specific TKIs in NSCLC cell lines (including H1975, HCC4006 and H1650) [36]–[39]. Acquired resistance has emerged as a major limitation of monotherapy with TKIs [40]. EGFR-targeted therapies can significantly improve disease control in EGFR-mutant patients, but the response is short-lived. EMT has recently been shown to have a role in acquired resistance to gefitinib in A549 cells, indicating that a mesenchymal phenotype is associated with the ‘inherent resistance’ to gefitinib or erlotinib in NSCLCs [41].

Our report shows that miR-147 recovered the EGFR-TKI gefitinib sensitivity of both colon cancer cell line HCT116 and lung cancer cell line A549 cells, which may be related to the inhibition of Akt and induction of other potential genes. For example, miR-147 inhibited EMP-1 gene expression by 6.4-fold (Table 1), which has been linked to the loss of sensitivity to gefitinib. EMP-1 was identified as a surface biomarker whose expression is correlated with the acquisition of gefitinib resistance [42]. It is also characterized as a mechanism of gefitinib resistance in 81B-Fb cells, where there was significantly more ligand (EGF)-induced phosphorylation of downstream Akt and Erk in the 81B-Fb cells that were more resistant to inhibition by gefitinib [43]. This is consistent with what our finding that miR-147 inhibited Akt phosphorylation. The activation of Akt through the ectopic expression of oncogenic PI3K or oncogenic Akt (myristoylated Akt) is sufficient to confer resistance to gefitinib, even in the presence of EGFR activating mutations. AKT and EMP-1, however, are likely only two of a number of genes affected by miR-147 that affect gefitinib resistance.

In our study, the expression of miR-147 induced MET and consequently reversed the native drug resistance of HCT116 associated with the “epithelial” EGFR inhibitor, gefitinib. Careful measurement of the expression of miR-147 and other miRs measured in FFPE tissues may provide a practical clinical test for predicting the response to drugs targeting the epithelial phenotype and may provide therapeutic approaches to increase drug sensitivity and combat resistance to the EGFR class of inhibitors.

## Conclusion

The ectopic expression of miR-147 was found to reverse the epithelial to mesenchymal transition, inhibit proliferation, induce G1 arrest and, reverse TGF-β1 induced EMT. Therefore, the expression miR-147 acted as a potential tumor suppressor. In addition, miR-147 dramatically reversed the intrinsic drug resistance associated with the “epithelial” EGFR inhibitor, gefitinib, in the colon cancer cell line HCT116. miR-147 significantly repressed Akt phosphorylation. Knockdown of Akt with siRNA did induce changes in the cell phenotype that were similar to those associated with the ectopic expression of miR-147. The striking increased sensitivity to gefitinib associated with the expression of miR-147 in addition to its cell cycle modulation suggests a new avenue for therapy.

## Supporting Information

Figure S1 **miR-147 induces a mesenchymal to epithelial transition (MET) in colon cancer cell lines HCT116.** (A) Negative control miR (miR-nc) transfected cells show a speculated and loosely connected mesenchymal phenotype (Original magnification of 100×). (B) miR-147 induces the cells a phenotype change to a more tightly associated rounded epithelial phenotype (Original magnification of 200×). The precursor miR-147 and negative control miR were transiently transfected into the cells, the phenotype changes noted two days after transfection.
(TIF)

Figure S2 **miR-147 induces a mesenchymal to epithelial transition (MET) in colon cancer cell lines SW480.** (A) miR-nc transfected SW480 cells are in loosely connected, spindle mesenchymal phenotype. (B) miR-147 transfected induced SW480 cells a more tightly associated rounded epithelial phenotype and many cells with big vacuoles (arrows). (Original magnification of 200×).
(TIF)

Figure S3 **miR-147 induces cell arrest in G1 phase in A549 cells.** The negative control miR transfected A549 cells show a normal cell cycle pattern (A), while the miR-147 transfected cells, show a dramatic reduction in S Phase (B). Cells transiently transfected with miR-147 and negative control miR, after 72 h post-transfection, DNA content was measured by flow cytometry to determine cell cycle fractions. Representative flow cytometric histograms of cells shown from three independent experiments. PI, propidium iodide.
(TIF)

Table S1 **miRNAs correlation to EMT signature scores on mean-centered data** **.**
(DOCX)

Table S2 **The number of gene expression changed over 2-fold by miR-147.** The analysis done by Affymetrix GeneChip U133 Plus2.0 platform. RNA from 5 isolated transfections of HCT116 cells transfected with miR-147 or miR-nc.
(DOCX)

## References

[1] Thiery JP, Sleeman JP (2006) Complex networks orchestrate epithelial-mesenchymal transitions. Nat Rev Mol Cell Biol 7: 131–142. 16493418 10.1038/nrm1835

[2] Lopez-Novoa JM, Nieto MA (2009) Inflammation and EMT: an alliance towards organ fibrosis and cancer progression. EMBO Mol Med 1: 303–314. 20049734 10.1002/emmm.200900043 PMC3378143

[3] Bartel DP (2004) MicroRNAs: genomics, biogenesis, mechanism, and function. Cell 116: 281–297. 14744438 10.1016/s0092-8674(04)00045-5

[4] Esquela-Kerscher A, Slack FJ (2006) Oncomirs - microRNAs with a role in cancer. Nat Rev Cancer 6: 259–269. 16557279 10.1038/nrc1840

[5] Xia H, Hui KM (2012) MicroRNAs involved in regulating epithelial-mesenchymal transition and cancer stem cells as molecular targets for cancer therapeutics. Cancer Gene Ther 19: 723–730. 22975591 10.1038/cgt.2012.58

[6] Gregory PA, Bert AG, Paterson EL, Barry SC, Tsykin A, et al (2008) The miR-200 family and miR-205 regulate epithelial to mesenchymal transition by targeting ZEB1 and SIP1. Nat Cell Biol 10: 593–601. 18376396 10.1038/ncb1722

[7] Korpal M, Lee ES, Hu G, Kang Y (2008) The miR-200 family inhibits epithelial-mesenchymal transition and cancer cell migration by direct targeting of E-cadherin transcriptional repressors ZEB1 and ZEB2. J Biol Chem 283: 14910–14914. 18411277 10.1074/jbc.C800074200 PMC3258899

[8] Park SM, Gaur AB, Lengyel E, Peter ME (2008) The miR-200 family determines the epithelial phenotype of cancer cells by targeting the E-cadherin repressors ZEB1 and ZEB2. Genes Dev 22: 894–907. 18381893 10.1101/gad.1640608 PMC2279201

[9] Harazono Y, Muramatsu T, Endo H, Uzawa N, Kawano T, et al (2013) miR-655 Is an EMT-suppressive MicroRNA targeting ZEB1 and TGFBR2. PLoS One 8: e62757. 23690952 10.1371/journal.pone.0062757 PMC3653886

[10] Bullock MD, Sayan AE, Packham GK, Mirnezami AH (2012) MicroRNAs: critical regulators of epithelial to mesenchymal (EMT) and mesenchymal to epithelial transition (MET) in cancer progression. Biol Cell 104: 3–12. 22188537 10.1111/boc.201100115

[11] Liu G, Friggeri A, Yang Y, Park YJ, Tsuruta Y, et al (2009) miR-147, a microRNA that is induced upon Toll-like receptor stimulation, regulates murine macrophage inflammatory responses. Proc Natl Acad Sci U S A 106: 15819–15824. 19721002 10.1073/pnas.0901216106 PMC2747202

[12] Uhlmann S, Mannsperger H, Zhang JD, Horvat EA, Schmidt C, et al (2012) Global microRNA level regulation of EGFR-driven cell-cycle protein network in breast cancer. Mol Syst Biol 8: 570. 22333974 10.1038/msb.2011.100 PMC3293631

[13] Loboda A, Nebozhyn MV, Watters JW, Buser CA, Shaw PM, et al (2011) EMT is the dominant program in human colon cancer. BMC Med Genomics 4: 9. 21251323 10.1186/1755-8794-4-9 PMC3032646

[14] Umbas R, Isaacs WB, Bringuier PP, Schaafsma HE, Karthaus HF, et al (1994) Decreased E-cadherin expression is associated with poor prognosis in patients with prostate cancer. Cancer Res 54: 3929–3933. 7518346

[15] Semb H, Christofori G (1998) The tumor-suppressor function of E-cadherin. Am J Hum Genet 63: 1588–1593. 9837810 10.1086/302173 PMC1377629

[16] Wellner U, Schubert J, Burk UC, Schmalhofer O, Zhu F, et al (2009) The EMT-activator ZEB1 promotes tumorigenicity by repressing stemness-inhibiting microRNAs. Nat Cell Biol 11: 1487–1495. 19935649 10.1038/ncb1998

[17] Brabletz S, Bajdak K, Meidhof S, Burk U, Niedermann G, et al (2011) The ZEB1/miR-200 feedback loop controls Notch signalling in cancer cells. EMBO J 30: 770–782. 21224848 10.1038/emboj.2010.349 PMC3041948

[18] Brabletz S, Brabletz T (2010) The ZEB/miR-200 feedback loop–a motor of cellular plasticity in development and cancer? EMBO Rep 11: 670–677. 20706219 10.1038/embor.2010.117 PMC2933868

[19] Ramaswamy S, Nakamura N, Vazquez F, Batt DB, Perera S, et al (1999) Regulation of G1 progression by the PTEN tumor suppressor protein is linked to inhibition of the phosphatidylinositol 3-kinase/Akt pathway. Proc Natl Acad Sci U S A 96: 2110–2115. 10051603 10.1073/pnas.96.5.2110 PMC26745

[20] Nawshad A, Lagamba D, Polad A, Hay ED (2005) Transforming growth factor-beta signaling during epithelial-mesenchymal transformation: implications for embryogenesis and tumor metastasis. Cells Tissues Organs 179: 11–23. 15942189 10.1159/000084505

[21] Miettinen PJ, Ebner R, Lopez AR, Derynck R (1994) TGF-beta induced transdifferentiation of mammary epithelial cells to mesenchymal cells: involvement of type I receptors. J Cell Biol 127: 2021–2036. 7806579 10.1083/jcb.127.6.2021 PMC2120317

[22] Kasai H, Allen JT, Mason RM, Kamimura T, Zhang Z (2005) TGF-beta1 induces human alveolar epithelial to mesenchymal cell transition (EMT). Respir Res 6: 56. 15946381 10.1186/1465-9921-6-56 PMC1177991

[23] Kosaka T, Yamaki E, Mogi A, Kuwano H (2011) Mechanisms of resistance to EGFR TKIs and development of a new generation of drugs in non-small-cell lung cancer. J Biomed Biotechnol 2011: 165214. 21687596 10.1155/2011/165214 PMC3114474

[24] Lamouille S, Derynck R (2007) Cell size and invasion in TGF-beta-induced epithelial to mesenchymal transition is regulated by activation of the mTOR pathway. J Cell Biol 178: 437–451. 17646396 10.1083/jcb.200611146 PMC2064840

[25] Sarbassov DD, Guertin DA, Ali SM, Sabatini DM (2005) Phosphorylation and regulation of Akt/PKB by the rictor-mTOR complex. Science 307: 1098–1101. 15718470 10.1126/science.1106148

[26] Wang X, Gorospe M, Huang Y, Holbrook NJ (1997) p27Kip1 overexpression causes apoptotic death of mammalian cells. Oncogene 15: 2991–2997. 9416843 10.1038/sj.onc.1201450

[27] Kwon TK, Nordin AA (1997) Overexpression of cyclin E and cyclin-dependent kinase inhibitor (p27Kip1): effect on cell cycle regulation in HeLa cells. Biochem Biophys Res Commun 238: 534–538. 9299546 10.1006/bbrc.1997.7335

[28] Li J, Yang XK, Yu XX, Ge ML, Wang WL, et al (2000) Overexpression of p27(KIP1) induced cell cycle arrest in G(1) phase and subsequent apoptosis in HCC-9204 cell line. World J Gastroenterol 6: 513–521. 11819639 10.3748/wjg.v6.i4.513 PMC4723549

[29] Baldin V, Lukas J, Marcote MJ, Pagano M, Draetta G (1993) Cyclin D1 is a nuclear protein required for cell cycle progression in G1. Genes Dev 7: 812–821. 8491378 10.1101/gad.7.5.812

[30] Xia W, Li J, Chen L, Huang B, Li S, et al (2010) MicroRNA-200b regulates cyclin D1 expression and promotes S-phase entry by targeting RND3 in HeLa cells. Mol Cell Biochem 344: 261–266. 20683643 10.1007/s11010-010-0550-2

[31] Scaltriti M, Baselga J (2006) The epidermal growth factor receptor pathway: a model for targeted therapy. Clin Cancer Res 12: 5268–5272. 17000658 10.1158/1078-0432.CCR-05-1554

[32] Alfieri RR, Galetti M, Tramonti S, Andreoli R, Mozzoni P, et al (2011) Metabolism of the EGFR tyrosin kinase inhibitor gefitinib by cytochrome P450 1A1 enzyme in EGFR-wild type non small cell lung cancer cell lines. Mol Cancer 10: 143. 22111840 10.1186/1476-4598-10-143 PMC3281800

[33] Birnbaum A, Ready N (2005) Gefitinib therapy for non-small cell lung cancer. Curr Treat Options Oncol 6: 75–81. 15610717 10.1007/s11864-005-0015-0

[34] Koizumi F, Kanzawa F, Ueda Y, Koh Y, Tsukiyama S, et al (2004) Synergistic interaction between the EGFR tyrosine kinase inhibitor gefitinib (“Iressa”) and the DNA topoisomerase I inhibitor CPT-11 (irinotecan) in human colorectal cancer cells. Int J Cancer 108: 464–472. 14648715 10.1002/ijc.11539

[35] Ware KE, Hinz TK, Kleczko E, Singleton KR, Marek LA, et al (2013) A mechanism of resistance to gefitinib mediated by cellular reprogramming and the acquisition of an FGF2-FGFR1 autocrine growth loop. Oncogenesis 2: e39. 23552882 10.1038/oncsis.2013.4 PMC3641357

[36] Thomson S, Petti F, Sujka-Kwok I, Epstein D, Haley JD (2008) Kinase switching in mesenchymal-like non-small cell lung cancer lines contributes to EGFR inhibitor resistance through pathway redundancy. Clin Exp Metastasis 25: 843–854. 18696232 10.1007/s10585-008-9200-4

[37] Frederick BA, Helfrich BA, Coldren CD, Zheng D, Chan D, et al (2007) Epithelial to mesenchymal transition predicts gefitinib resistance in cell lines of head and neck squamous cell carcinoma and non-small cell lung carcinoma. Mol Cancer Ther 6: 1683–1691. 17541031 10.1158/1535-7163.MCT-07-0138

[38] Suda K, Tomizawa K, Fujii M, Murakami H, Osada H, et al (2011) Epithelial to mesenchymal transition in an epidermal growth factor receptor-mutant lung cancer cell line with acquired resistance to erlotinib. J Thorac Oncol 6: 1152–1161. 21597390 10.1097/JTO.0b013e318216ee52

[39] Yao Z, Fenoglio S, Gao DC, Camiolo M, Stiles B, et al (2010) TGF-beta IL-6 axis mediates selective and adaptive mechanisms of resistance to molecular targeted therapy in lung cancer. Proc Natl Acad Sci U S A 107: 15535–15540. 20713723 10.1073/pnas.1009472107 PMC2932568

[40] Glickman MS, Sawyers CL (2012) Converting cancer therapies into cures: lessons from infectious diseases. Cell 148: 1089–1098. 22424221 10.1016/j.cell.2012.02.015 PMC3465702

[41] Rho JK, Choi YJ, Lee JK, Ryoo BY, Na II, et al (2009) Epithelial to mesenchymal transition derived from repeated exposure to gefitinib determines the sensitivity to EGFR inhibitors in A549, a non-small cell lung cancer cell line. Lung Cancer 63: 219–226. 18599154 10.1016/j.lungcan.2008.05.017

[42] Jain A, Tindell CA, Laux I, Hunter JB, Curran J, et al (2005) Epithelial membrane protein-1 is a biomarker of gefitinib resistance. Proc Natl Acad Sci U S A 102: 11858–11863. 16087880 10.1073/pnas.0502113102 PMC1187965

[43] Maseki S, Ijichi K, Tanaka H, Fujii M, Hasegawa Y, et al (2012) Acquisition of EMT phenotype in the gefitinib-resistant cells of a head and neck squamous cell carcinoma cell line through Akt/GSK-3beta/snail signalling pathway. Br J Cancer 106: 1196–1204. 22315058 10.1038/bjc.2012.24 PMC3304404

