# Supplementary figures and images for: MicroRNA-147 Induces a Mesenchymal-To-Epithelial Transition (MET) and Reverses EGFR Inhibitor Resistance
Source: PLoS One. 2014 Jan 15;9(1):e84597. doi: 10.1371/journal.pone.0084597 (PMC3893127; doi:10.1371/journal.pone.0084597)

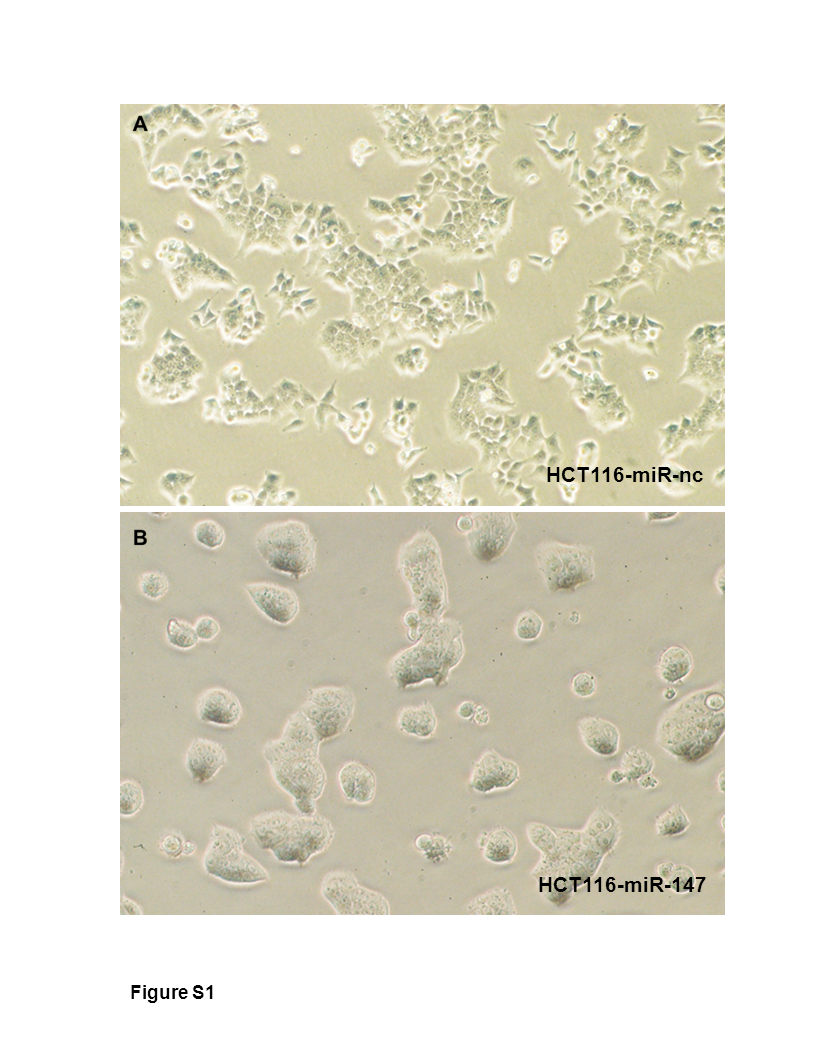

Supplement: Figure S1 — miR-147 induces a mesenchymal to epithelial transition (MET) in colon cancer cell lines HCT116. (A) Negative control miR (miR-nc) transfected cells show a speculated and loosely connected mesenchymal phenotype (Original magnification of 100×). (B) miR-147 induces the cells a phenotype change to a more tightly associated rounded epithelial phenotype (Original magnification of 200×). The precursor miR-147 and negative control miR were transiently transfected into the cells, the phenotype changes noted two days after transfection. (TIF) [file pone.0084597.s001.tif]

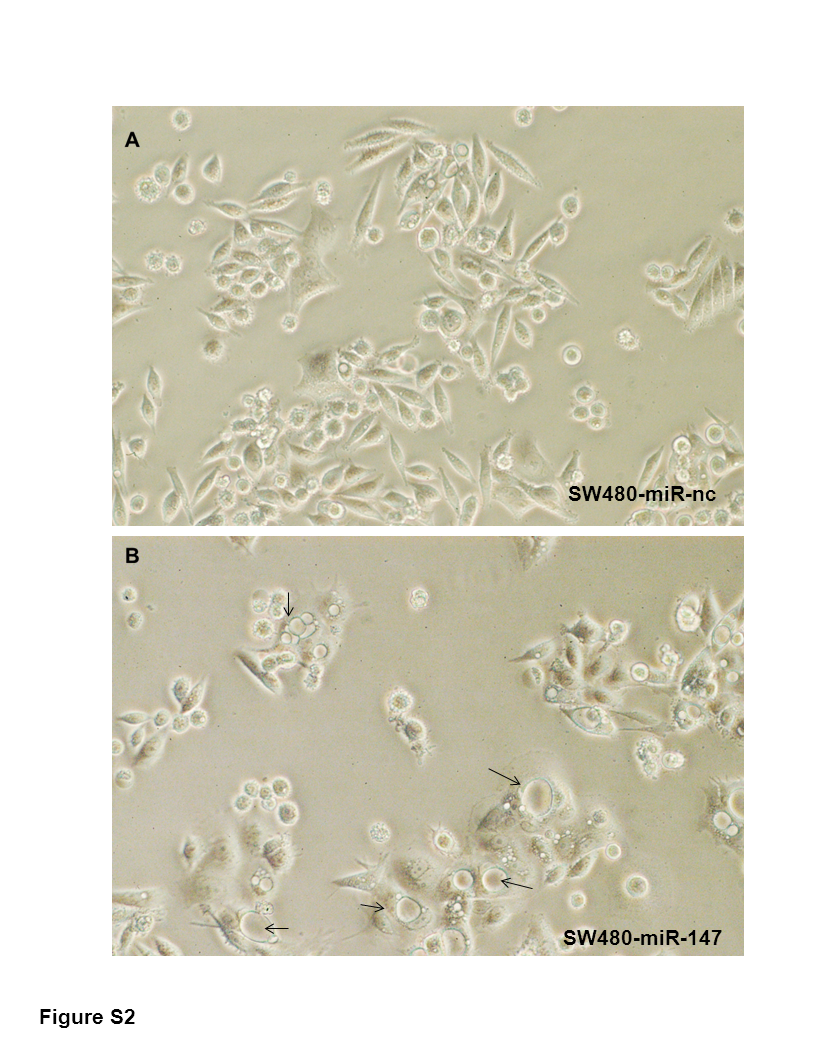

Supplement: Figure S2 — miR-147 induces a mesenchymal to epithelial transition (MET) in colon cancer cell lines SW480. (A) miR-nc transfected SW480 cells are in loosely connected, spindle mesenchymal phenotype. (B) miR-147 transfected induced SW480 cells a more tightly associated rounded epithelial phenotype and many cells with big vacuoles (arrows). (Original magnification of 200×). (TIF) [file pone.0084597.s002.tif]

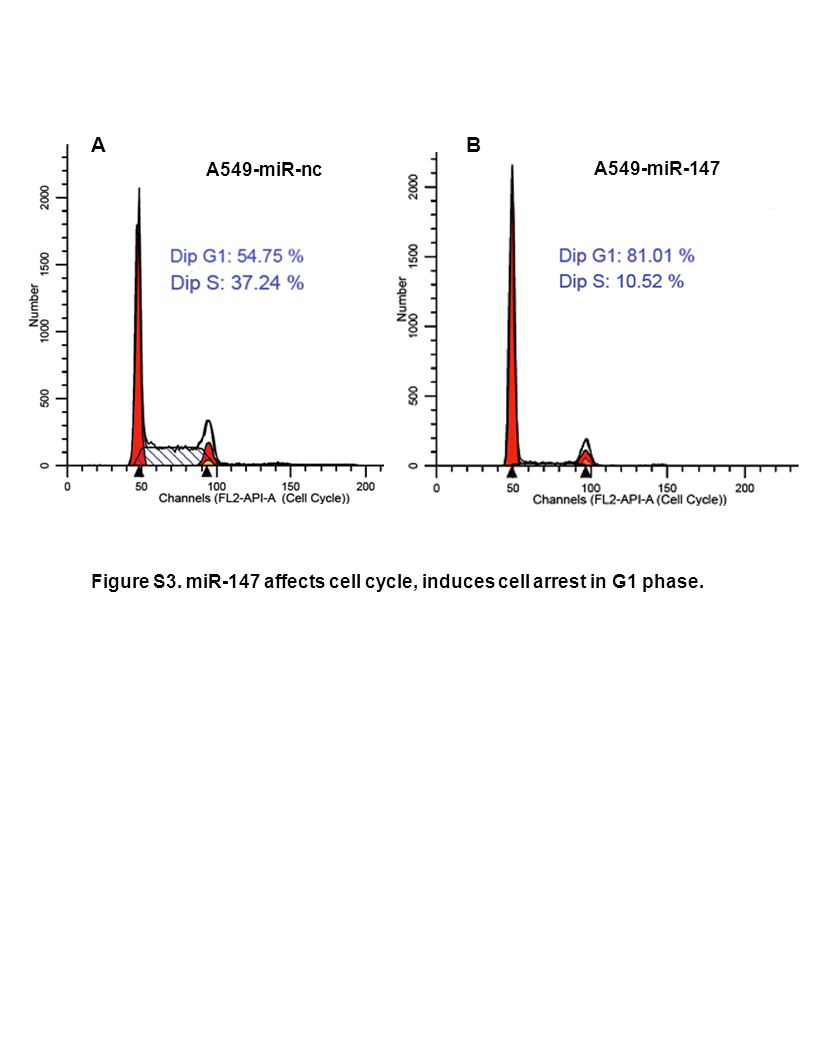

Supplement: Figure S3 — miR-147 induces cell arrest in G1 phase in A549 cells. The negative control miR transfected A549 cells show a normal cell cycle pattern (A), while the miR-147 transfected cells, show a dramatic reduction in S Phase (B). Cells transiently transfected with miR-147 and negative control miR, after 72 h post-transfection, DNA content was measured by flow cytometry to determine cell cycle fractions. Representative flow cytometric histograms of cells shown from three independent experiments. PI, propidium iodide. (TIF) [file pone.0084597.s003.tif]

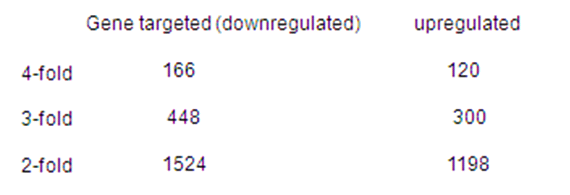


**Table S2. The number of genes changed over 2-fold by miR-147.**

Supplement: Table S2 — The number of gene expression changed over 2-fold by miR-147. The analysis done by Affymetrix GeneChip U133 Plus2.0 platform. RNA from 5 isolated transfections of HCT116 cells transfected with miR-147 or miR-nc. (DOCX) [file pone.0084597.s005.docx]
