# Supplementary material for: MicroRNA-147 Induces a Mesenchymal-To-Epithelial Transition (MET) and Reverses EGFR Inhibitor Resistance
Source: PLoS One. 2014 Jan 15;9(1):e84597. doi: 10.1371/journal.pone.0084597 (PMC3893127; doi:10.1371/journal.pone.0084597)
Supplement: Table S1 — miRNAs correlation to EMT signature scores on mean-centered data . (DOCX) [file pone.0084597.s004.docx]

| miRs EMT corr p-value |
| --- |
| Has-miR-519a-4395526 (FAM, NFQ ) -25% 8.E02  Has-miR- 922-4395263 (FAM, NFQ ) -25% 8.E02  Has-miR-645-4381000 (FAM, NFQ ) -27% 6.E02  Has-miR-512-3p-4381034 (FAM, NFQ ) -27% 6.E02  Has-miR-148a-4373130 (FAM, NFQ ) -28% 5.E02  Has-miR-425-4380926 (FAM, NFQ ) -28% 5.E02  Has-miR-148b-4373129 (FAM, NFQ ) -29% 4.E02  Has-miR-449b-4381011 (FAM, NFQ ) -30% 4.E02  Has-miR-147-4373131 (FAM, NFQ ) -31% 3.E02  Has-miR-875-5p-1395314 (FAM, NFQ ) -33% 2E02  Has-miR-147b-4395373 (FAM, NFQ ) -34% 2.E02  Has-miR- 942-4395298 (FAM, NFQ ) -34% 2.E02  Has-miR-885-5p-4395207 (FAM, NFQ ) -35% 1.E02  Has-miR-517a-4395513 (FAM, NFQ ) -39% 6.E02  Has-miR-576-3p-4395462 (FAM, NFQ ) -39% 6.E02 |

**Table S1**. **miRNAs correlation to EMT signature scores on mean-centered data.**
